# Contactless Inductive Flow Tomography for Real-Time Control of Electromagnetic Actuators in Metal Casting

**DOI:** 10.3390/s22114155

**Published:** 2022-05-30

**Authors:** Ivan Glavinić, Vladimir Galindo, Frank Stefani, Sven Eckert, Thomas Wondrak

**Affiliations:** Department of Magnetohydrodynamics, Institute of Fluid Dynamics, Helmholtz-Zentrum Dresden-Rossendorf, Bautzner Landstraße 400, 01328 Dresden, Germany; v.galindo@hzdr.de (V.G.); f.stefani@hzdr.de (F.S.); s.eckert@hzdr.de (S.E.); t.wondrak@hzdr.de (T.W.)

**Keywords:** contactless inductive flow tomography, continuous casting, flow monitoring, EMBr, inductive measurements, mini-LIMMCAST

## Abstract

Flow control of liquid metals based on the actual flow condition is important in many metallurgical applications. For instance, the liquid steel flow in the mould of a continuous caster strongly influences the product quality. The flow can be modified by an electromagnetic brake (EMBr). However, due to the lack of appropriate flow measurement techniques, the control of those actuators is usually not based on the actual flow condition. This article describes the recent developments of the Contactless Inductive Flow Tomography (CIFT) towards a real-time monitoring system, which can be used as an input to the control loop for an EMBr. CIFT relies on measuring the flow-induced perturbation of an applied magnetic field and the solution of an underlying linear inverse problem. In order to implement the CIFT reconstructions in combination with EMBr, two issues have to be solved: (i) compensation of the effects of the change in EMBr strength on the CIFT measurement system and (ii) a real-time solution of the inverse problem. We present solutions of both problems for a model of a continuous caster with a ruler-type EMBr. The EMBr introduces offsets of the measured magnetic field that are several orders of magnitude larger than the very flow-induced perturbations. The offset stems from the ferromagnetic hysteresis exhibited by the ferrous parts of the EMBr in the proximity of the measurement coils. Compensation of the offset was successfully achieved by implementing a numerical model of hysteresis to predict the offset. Real-time reconstruction was achieved by precalculating the computationally heavy matrix inverses for a predefined set of regularization parameters and choosing the optimal one in every measurement frame. Finally, we show that this approach does not hinder the reconstruction quality.

## 1. Introduction

Continuous casting is a step in the modern steel-making process in which the production transitions from the batch process to a continuous one. Batches of liquid steel are brought by ladles from the secondary metallurgy process. The liquid steel is then drained into the tundish, which acts as a buffer storage between ladle changes. From the tundish, the liquid steel flows through the Submerged Entry Nozzle (SEN) into the water-cooled mould. The flow rate through the SEN is controlled by the position of the stopper rod at the SEN inlet or via a sliding gate located in the upper SEN area. A continuous process is achieved by constantly extruding the partly solidified product from the mould. The solid shell, with a still liquid core, is then guided by a series of support rolls and actively cooled until the product is fully solidified. The long strand of solid steel is cut to the desired lengths and prepared for subsequent processes. The shape of the end product is determined by the mould profile. In this work, we focus on the production of rectangular slabs of steel.

The flow in the mould has a major effect on the quality of the produced steel. Typically, two different flow patterns can be distinguished: a single-roll flow and a double-roll flow. In general, the double-roll flow is considered more desirable as it is correlated with fewer defects [1]. The key challenge is controlling the optimal flow velocity on the meniscus. Too high velocities promote the entrainment of liquid slag into the liquid steel, generating inclusions. However, if the meniscus velocities are too low, the liquid steel at the meniscus cools down and starts to solidify, especially close to the narrow face of the mould, and a hook may form on which the slag can attach, leading to surface defects [2].

The flow in the mould can be influenced by electromagnetic actuators. For example, an electromagnetic brake (EMBr) generates a stationary magnetic field which induces Lorentz forces that alter the flow structure and usually dampen the flow [3]. However, under certain circumstances, flow acceleration can also occur [4]. Electromagnetic stirrers (EMS) use a rotating or a travelling magnetic field to induce the movement of the liquid metal. In this article, we focus on the application of a ruler type EMBr for slab casting moulds. As a rule, the field strength of the EMBr is specified by a casting recipe, which is based on certain assumptions and empirical values and is not controlled on the basis of the actual flow state in the mould. This is mainly due to the fact that there are no suitable flow measurement methods.

High temperatures and the chemical aggressiveness of liquid steel limit the applicability and performance of suitable measuring methods. The most recent measurement system that has become available measures the two-dimensional temperature distribution at the wide and narrow faces of the mould by using mould-embedded fiber Bragg gratings [5,6]. From the temperature maps, the meniscus profile can be determined, and some general conclusions on the flow structure in the mould can be drawn. A successful approach of controlling the strength of an EMBr for a thin caster based on temperature maps was recently proposed [7]. However, a direct inference of the entire flow field from those temperature maps remains elusive.

The meniscus velocity of the liquid steel can be measured by inserting a paddle and measuring the inclination angle or by the nail-board technique [8]. These techniques require direct contact with the liquid steel and are not suitable for long-lasting measurements. Contactless local flow measurements have been conducted with AMEPA’s Mould Flow Measurement [8,9], where a near-wall velocity is derived from the correlation of fluctuating signals recorded by two inductive sensors embedded in the mould wall. Another attempt for monitoring the meniscus velocities was made by using Lorentz Force Velocimetry [10]. A measurement technique for monitoring the complete flow structure in the mould is not available yet.

A promising candidate in this respect is the Contactless Inductive Flow Tomography (CIFT), which aims at resolving the full three-dimensional flow structure of an electrically conductive fluid [11]. The working principle is based on measuring the flow-induced perturbations of one, or several, externally applied magnetic field(s). The adaptation of CIFT for continuous casting was investigated on a small scale laboratory model with a scaling of about 1:8, where time-dependent dominant two-dimensional flow structures were successfully monitored [12,13]. The scalability of this technique was demonstrated later by successful reconstructions of the flow field in the mould of a large scale 1:2 laboratory model [14].

A key challenge of CIFT is measuring the small flow-induced perturbation of the applied magnetic field. Typically, for an applied magnetic field with a strength of about 1 mT, the perturbations are in the order of several nT. Because of this dynamic range of six orders of magnitude, the measurement technique is susceptible to environmental noise. However, great improvement in the robustness of the measurement technique has been achieved with the deployment of gradiometric coils [15,16] and by utilizing an AC excitation magnetic field and locking in on the set frequency. When using AC excitation, the measured signal is demodulated, and the information of the flow-induced magnetic field is encoded in the amplitude of the signal in-phase to the excitation magnetic field [17]. With these techniques, it became possible to apply CIFT even in the presence of an EMBr that generates a static magnetic field of about 300 mT. Here, it was shown that the alternation of the flow structure by the EMBr can be safely identified by CIFT [18,19].

In order to implement any control strategies for an EMBr, the CIFT measurement system has to cope with the changes in EMBr strength during the measurement. Preliminary work showed a strong influence on the CIFT measurements but also indicated that a compensation of the EMBr with respect to the measured magnetic field is possible [20,21,22]. The compensation is particularly challenging due to the deformation of the applied CIFT excitation field due to the non-linear behaviour of the ferromagnetic parts of the EMBr, which we will discuss in detail later in Section 3.2.

In this article, we present a study of the influence of an EMBr on CIFT measurements. We show possibilities and strategies of how undesired effects can be compensated. We introduce the real-time reconstruction algorithm and analyse its accuracy. The experiments were conducted at the laboratory model of a continuous caster (mini-LIMMCAST) located at Helmholtz-Zentrum Dresden-Rossendorf (HZDR).

After giving a short explanation of the CIFT basics (Section 2.1) and the mini-LIMMCAST facility (Section 2.2), we present two different compensation strategies (Section 3.2). Finally, we describe the real-time algorithm for the solution of the inverse problem, which enables the implementation of a real-time CIFT monitoring system (Section 4).

## 2. Materials and Methods

### 2.1. Contactless Inductive Flow Tomography

The operation principle of CIFT is based on the fact that a moving electrically conductive fluid perturbs an external magnetic field. The flow-induced change of the magnetic field contains the information about the space-time structure of the flow, so the velocity field can be reconstructed by magnetic field measurement and the solution of the corresponding inverse mathematical problem. Considered is a fluid with a homogeneous conductivity σ and the velocity field v in volume *V* that is under the influence of the magnetic field B. The interaction between magnetic field and fluid flow produces an electromotive force which drives a current j in the fluid according to Ohm’s law:

(1) $$j=\sigma \left(v\times B-\nabla \varphi \right).$$

From Biot–Savart’s law a secondary magnetic field at the position r outside the fluid volume *V* can be calculated from the contributions of current at the position $r{}^{\prime }$ within the fluid volume

(2) $$b\left(r\right)=\frac{\mu_{0}}{4\pi }{∭}_{V}j\left(r{}^{\prime }\right)\times \frac{r-r{}^{\prime }}{{\left|r-r{}^{\prime }\right|}^{3}}dV{}^{\prime },$$

where $\mu_{0}$ is the magnetic permeability of vacuum. Due to charge conservation, the divergence of the current is zero:

(3) ∇·j=0.

Then, from Equation (1), we obtain the Poisson equation for the electric potential φ:

(4) $$\nabla^{2}\varphi =\nabla \left(v\times B\right).$$

By substituting Equation (1) into Equation (2) and applying Gauss’ theorem to express the potential term as the surface integral over the fluid boundary, and by resolving Equation (4), we obtain the following system of integral equations that needs to be inverted to determine the velocity v from the measurement of the secondary magnetic fields b at the points r outside the volume *V*:

(5) $$b\left(r\right)=\frac{\mu_{0}\sigma }{4\pi }{∭}_{V}\frac{\left(v\left(r{}^{\prime }\right)\times B\left(r{}^{\prime }\right)\right)\times \left(r-r{}^{\prime }\right)}{|r-r{}^{\prime }{|}^{3}}dV^{\prime }-\frac{\mu_{0}\sigma }{4\pi }{∯}_{S}\frac{\varphi \left(r{}^{\prime }\right)n\left(r{}^{\prime }\right)\times \left(r-r{}^{\prime }\right)}{|r-r{}^{\prime }{|}^{3}}dS^{\prime },$$

(6) $$p\left(r\right)\varphi \left(r\right)=\frac{1}{4\pi }{∭}_{V}\frac{\left(v\left(r{}^{\prime }\right)\times B\left(r{}^{\prime }\right)\right)\cdot \left(r-r{}^{\prime }\right)}{|r-r{}^{\prime }{|}^{3}}dV^{\prime }-\frac{1}{4\pi }{∯}_{S}\frac{\varphi \left(r{}^{\prime }\right)n\left(r{}^{\prime }\right)\cdot \left(r-r{}^{\prime }\right)}{|r-r{}^{\prime }{|}^{3}}dS^{\prime }.$$

Here, p(r) is a factor, between 0≤p(r)≤1, determined by the shape of the boundary which depends on the solid angle of the surface at the position r. $dV^{\prime }$ and $dS^{\prime }$ represent volume and surface elements, respectively. B(r) in general is the sum of the applied excitation magnetic field $B_{0}\left(r\right)$ (the primary field) and the flow-induced magnetic field b(r) (the secondary field). As in our application, the ratio of flow-induced magnetic field and the applied magnetic field, defined by the magnetic Reynolds number

(7) $$R_{m}=vl\mu_{0}\sigma ,$$

is well below 1 (given that the characteristic velocity *v* is the inlet velocity of 1.4 m/s, the typical length scale *l* is the diameter of the jet of 15 mm, and the conductivity σ of GaInSn is 3.3 MS/m [12,22]), we can ignore the effect of the flow-induced magnetic field on the velocity field and substitute B(r) in Equations (5) and (6) with $B_{0}\left(r\right)$, resulting in a linear problem in v. The system of integral equations is solved numerically by discretizing the fluid domain and applying linear shape functions to the resulting volume and surface elements, yielding the following linear equation:

(8) $$C\cdot \tilde{v}=\tilde{b}.$$

The vector $\tilde{b}\in R^{n}$ contains the values of all measured magnetic field perturbations at *n* sensor locations. The system matrix $C\in R^{n\times 3m}$ calculates the magnetic field at the sensors in dependence of the given velocity field $\tilde{v}\in R^{3m}$ in the fluid volume at *m* nodes of the mesh. For the application of continuous casting, an additional constraint on the inlet velocity is introduced. This is completed by concatenating the system matrix with the inlet velocity selection sparse matrix $E\in R^{h\times 3m}$ and the measurement vector with values of inlet velocity $v_{inlet}$ at the *h* inlet nodes:

(9) $$\bar{C}=\left[\begin{array}{c} C(B_{0})\\ E \end{array}\right],\;\bar{b}=\left[\begin{array}{c} \tilde{b}\\ v_{inlet} \end{array}\right].$$

In order to reconstruct the velocity field $\tilde{v}$, the following minimization problem has to be resolved with respect to $\tilde{v}$:

(10) $$\min_{\tilde{v}}\left({∥\bar{C}\cdot \tilde{v}-\bar{b}∥}_{2}^{2}+\lambda_{L}{∥L\cdot \tilde{v}∥}_{2}^{2}+\lambda_{G}{∥G\cdot \tilde{v}∥}_{2}^{2}+\lambda {∥D\cdot \tilde{v}∥}_{2}^{2}\right).$$

The first term represents the residuum of the calculated and measured flow-induced magnetic field. The matrix L calculates the Laplacian of the flow, and the matrix G calculates the divergence of the velocity field. D is the matrix for Tikhonov regularization. The parameter λ balances the minimum of the residuum and the minimum kinetic velocity of the flow, and it is determined by the L-curve method [23].

In comparison to the previous experiments by Ratajczak et al. [18], our mould geometry is twice as wide, and the sensors are further away from the SEN outlet. Because the sensors are only located on the narrow sides of the mould, there is a significant part of the geometry between the SEN outlet and the narrow faces of the mould where the velocity is difficult to reconstruct. We tried also to mitigate this by introducing the Laplacian operator L in the regularization in order to constrain the rate of change of the velocity field but without obtaining noticeable improvements. A more detailed description of the solution of the inverse problem is given in Section 4.

### 2.2. Mini-LIMMCAST

**Figure 1:**
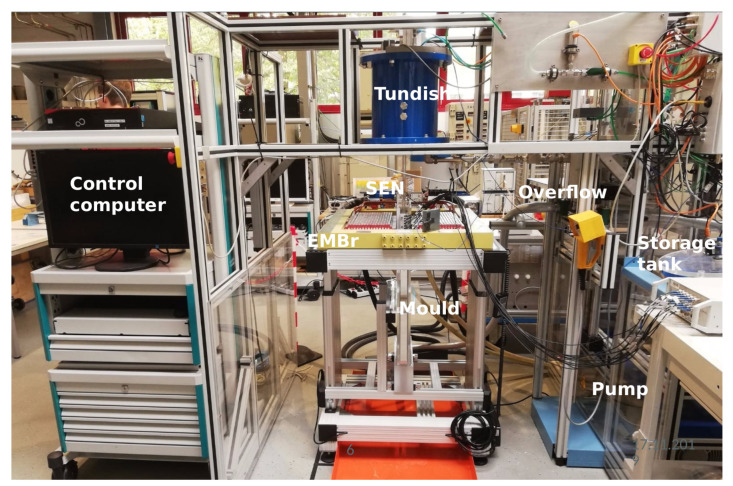
Photograph of mini-LIMMCAST laboratory model.

Experiments were conducted using mini-LIMMCAST, an isothermal laboratory model of a continuous caster that is operated with the eutectic non-toxic alloy gallium-indium-tin (GaInSn) at room temperature. Figure 1 shows a photograph of the mini-LIMMCAST facility. Liquid metal is pumped from the storage tank to the tundish. From the tundish, liquid metal flows into the mould through the SEN. The mould has a rectangular profile of 35 × 300 mm^2^. The SEN has an inner diameter of 12 mm and an outer diameter of 21 mm with two side ports directed downwards at an angle of 15°.

The experimental setup is equipped with an EMBr to exert a Lorentz force on the flow. The effect of Lorentz’s force on the fluid alters the flow pattern in the mould, and based on the position and strength of the EMBr, the jet changes its shape and its impingement position at the narrow faces of the mould. The EMBr can generate a magnetic field up to 400 mT with the electric current of 600 A. With selected mould dimensions, reasonable Reynolds and Hartmann similarities are achieved for the 1:3 to 1:4 scale of an industrial continuous caster [4].

**Figure 2:**
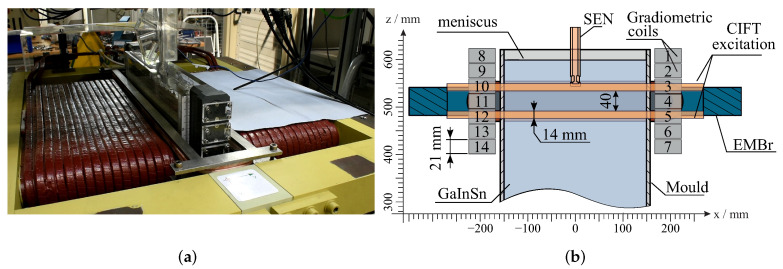
Experimental setup comprising CIFT coils and sensors as well as the electromagnetic brake (EMBr). Two excitation coils generate a primarily vertical magnetic field. Fourteen gradiometric coils, seven on each narrow side, are used to measure the flow-induced magnetic field. The EMBr generates a strong magnetic field below the submerged entry nozzle (SEN), perpendicular to the wide side of the mould. (**a**) Photograph of the mould and CIFT coils. (**b**) Sketch adopted from Schurmann et al. [4].

The mould is equipped with the CIFT setup, as shown in Figure 2. It consists of two excitation coils, one above and one below the ferromagnetic yoke of the EMBr, that generate an excitation magnetic field of 1.5 mT. To measure the flow-induced magnetic field, fourteen gradiometric coils are used, with seven on each narrow side of the mould.

Using induction coils for magnetic measurement has several advantages compared to other methods. Induction coils are insensitive to static magnetic fields, which is important when detecting flow-induced magnetic fields in the presence of the strong static magnetic field of the EMBr. Additionally, induction coils have a wide measurement range and a high sensitivity. However, to achieve the desired sensitivity for the minuscule flow-induced magnetic field, a large number of turns of thin wire are needed, resulting in a high impedance, requiring special A/D converters with high input resistance. The employed gradiometric coils, consisting of two coils wound in opposite directions and connected in series are largely unaffected by variable magnetic fields that are uniform along the sensor axis, such as the Earth’s magnetic field [16].

## 3. Compensation for the Impact of the EMBr on the CIFT Measurement System

Because an AC magnetic field with frequency of f= 8 Hz is used, the information about the flow is encoded in the in-phase component of the magnetic field measured by the gradiometric coils. To extract the flow-induced magnetic field, we first measure the in-phase component of the applied magnetic field, undisturbed by the flow, at the beginning of the experiment (*t* = 0 s) and subtract this offset from the consecutive magnetic field measurements. The source of any change of the measured in-phase value is assumed to stem from the flow-induced magnetic field.

**Figure 3:**
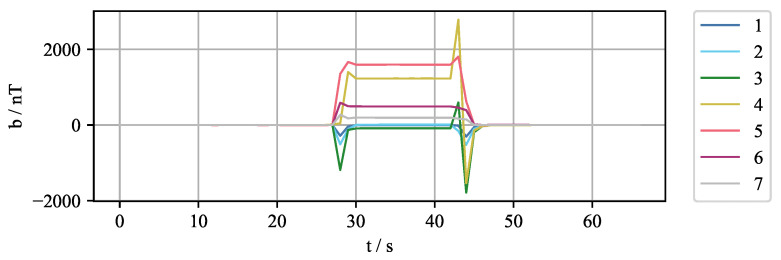
Measurement of the magnetic field for an experiment where the EMBr current $I_{B}$ is changed during the measurement for the sensors 1–7. The current was turned on at t≈ 28 s to $I_{B}$ = 200 A and turned off at t≈44 s to $I_{B}$ = 0 A.

However, when the strength of the EMBr changes, the magnetic properties of the ferromagnetic parts also change, resulting in the deformation of the applied magnetic field. This change is also detected by the sensors and is sometimes even one to two orders of magnitude larger than the expected flow-induced magnetic field, as is visible in Figure 3. It shows the effects of switching the EMBr to a current of 200 A and switching the EMBr off for the case without the liquid metal flow. It is evident that sensors 4 and 5, which are closest to the ferromagnetic part (see also Figure 2b), exhibit a change of more than 1000 nT, which is two orders of magnitude larger than the expected flow-induced magnetic field.

The underlying cause of the deformation of the excitation magnetic field is due to the change in the magnetic properties of the ferromagnetic parts. When the current is applied to the EMBr, the magnetic field $H_{B}$ changes accordingly. This change of the magnetic field has an influence on the bulk magnetization within the ferromagnetic yoke of the EMBr, which can be expressed as a function of the magnetic field

(11) $$M=f(H_{B})$$

where M is the magnetization vector. The total EMBr magnetic flux density $B_{B}$ is given by

(12) $$B_{B}=\mu_{0}\left(H_{B}+M\right)$$

If we consider that the CIFT excitation magnetic field $H_{0}$ is closing through the same ferromagnetic domain, it can be assumed that the magnetic field flux density $B_{0}$ also changes as a function of magnetization M. The result is a sudden and significant offset of the in-phase component of the measured magnetic field when the EMBr is switched on. The measured offset remains constant over the entire period because the EMBr current is kept constant.

**Figure 4:**
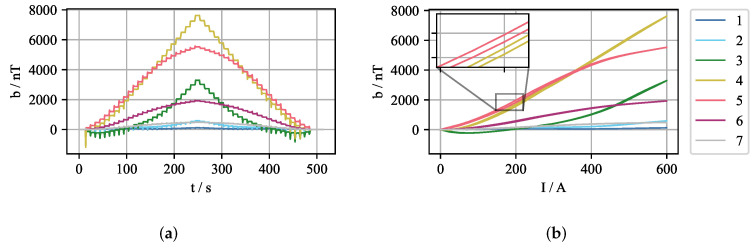
Measured offset of the in-phase component of the magnetic field for an experiment where the EMBr current was cycled from 0 A to 600 A and back to 0 A in steps of $\Delta I_{B}$ = 25 A. The plots show only measurements for sensors 1–7. (**a**) plot over time; (**b**) plot of offset over EMBr current where the hysteresis can be observed.

In order to investigate this behaviour in more detail, the EMBr current $I_{B}$ was cycled up to 600 A and back to 0 A in steps of $\Delta I_{B}$ = 25 A. For each current step, the average offset of the in-phase component of the magnetic field was measured. Figure 4 shows the plot of the current over time and current-offset pairs where the slight hysteresis is visible.

Clearly, the effect is not negligible and has to be compensated during the measurement. Additionally, the deformation of the applied magnetic field depends not only on the current state but also on the history of changes, which comes from the underlying property of the ferromagnetic hysteresis exhibited by the parts of the EMBr.

### 3.1. Krasnosel’skii–Pokrowski Model of Hysteresis

This section introduces the hysteresis model that is selected to compensate for such effects. Although several models describe the hysteresis phenomenon well, we opted for the Krasnosel’skii–Pokrowski (KP) model, which is an extension of the Preisach hysteresis model. The main difference from the Preisach model is that the relay operator is replaced with an operator based on Krasnosel’skii–Pokrowski’s notion of generalized relays [24,25]. The KP model was chosen because it can model the asymmetric hysteresis that was observed in the initial experiments. Alternatively, a modified Prandtl–Ishlinskii model could also model the asymmetric hysteresis. However, it does not satisfy the congruency property on which we base one of our compensation methods [26]. Differential equation based models, such as the Bouc-Wen and Duhem model, could also be used. Nonetheless, here we opted to use the KP model for its simpler implementation. The usual downsides associated with operator-based models, such as the model size and the computational performance, do not play a huge role in this setup because the time sampling rate of the model input is in the order of seconds, and the input is limited to only 24 discrete values.

The hysteresis model is defined as a linear combination of the weighted KP $k_{p}$ operators

(13) $$b\left(t\right)=\iint_{P}k_{p}\left(I_{B}\left(t\right),\xi_{p}\left(t\right)\right)\mu_{p}dp,$$

where b(t) is the output value of the magnetic field change for the input currents $I_{B}\left(t\right)$. $k_{p}\left(I_{B}\left(t\right),\xi_{p}\left(t\right)\right)$ is an elementary KP operator on the position *p* of the Preisach plane. $\xi_{p}\left(t\right)$ models the memory of the hysteresis and $\mu_{p}$ is the weight function that determines the shape of the hysteresis. *P* is the Preisach plane, defined as a set of all admissible pairs *p* of thresholds $p_{1}$ and $p_{2}$ of the kernel operators

(14) $$P=\{p=\left(p_{1},p_{2}\right)\in R^{2}:I_{B,\;min}\leq p_{1}\leq p_{2}\leq I_{B,\;max}\}.$$

The KP operator is defined as:

(15) $$k_{p}\left(I_{B}\left(t\right),\xi_{p}\left(t\right)\right)=\left\{\begin{array}{cc} \max\{\xi_{p}\left(t\right),r\left(I_{B}\left(t\right)-p_{2}\right)\} & \mathrm{if}\;dI_{B}/dt\geq 0\\ \min\{\xi_{p}\left(t\right),r\left(I_{B}\left(t\right)-p_{1}\right)\} & \mathrm{if}\;dI_{B}/dt<0 \end{array}\right.$$

where $\xi_{p}\left(t\right)$ represents the initial state of the system as well as the memory of the last operator output extrema for an operator $k_{p}$ at the time *t* and is defined as

(16) $$\xi_{p}\left(t\right)=\left\{\begin{array}{cc} 0 & t=t_{0}\\ k_{p}\left(I_{B}\left(t_{i}\right),\xi_{p}\left(t_{i-1}\right)\right) & t>t_{0};\;sign{\left(dI_{B}/dt\right)}_{t_{i}}=-sign{\left(dI_{B}/dt\right)}_{t_{i-1}}\\ \xi_{p}\left(t_{i-1}\right) & t>t_{0};\;sign{\left(dI_{B}/dt\right)}_{t_{i}}=sign{\left(dI_{B}/dt\right)}_{t_{i-1}}. \end{array}\right.$$

The ridge function *r* is defined as

(17) $$r\left(x\right)=\left\{\begin{array}{cc} -1 & x<0\\ -1+\frac{2x}{a} & 0\geq x\geq a\\ 1 & x>a \end{array}\right.$$

where *a* is a heuristically selected value which determines the slope of the KP operator.

**Figure 5:**
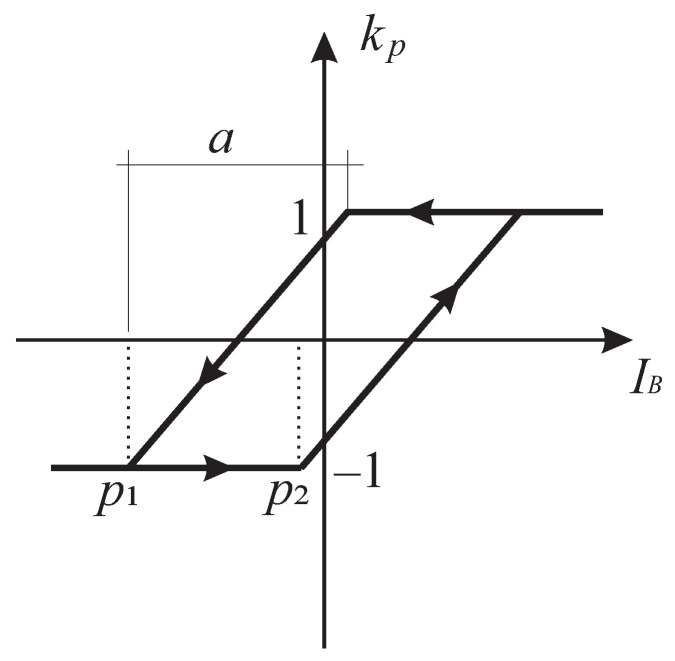
KP operator.

Figure 5 represents the plot of one kernel operator as defined by Equation (15) at an arbitrary location ($p_{1}$, $p_{2}$) on the Preisach plane for an input $I_{B}\left(t\right)$ that is increasing and subsequently decreasing over time. Let us assume that the input $I_{B}\left(t_{0}\right)$ starts with an initial value far less than the rising threshold $p_{2}$ and increases continuously over time *t*. As long as $I_{B}\left(t\right)$ is smaller than $p_{2}$, the KP operator outputs −1. When $I_{B}\left(t\right)$ increases further, the output of the KP operator starts increasing and reaches 1 when $I_{B}\left(t\right)=p_{2}+a$ is satisfied. If $I_{B}\left(t\right)$ increases even more, the output is not changed. In case the input $I_{B}\left(t\right)$ starts to decrease, the output of the KP operator is 1, until $I_{B}\left(t\right)=p_{1}+a$. Further decrease in the input value starts to decrease the output and reaches -1 when the input is $I_{B}\left(t\right)=p_{1}$.

**Figure 6:**
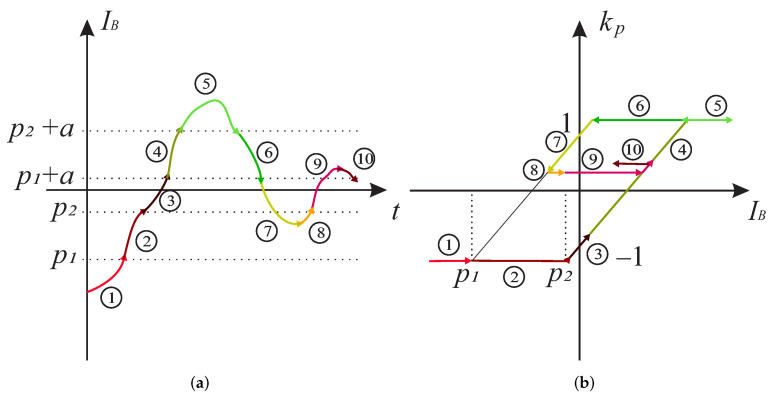
Generalized output of a single KP operator. (**a**) Input function $I_{B}\left(t\right)$ in relation to parameters $p_{1}$, $p_{2}$ and *a*. (**b**) Generalized output of the KP operator for the given input function. Colours represent the values of the input and the corresponding output of the KP operator.

In order to give an illustrative example, Figure 6a shows an arbitrary time dependent current $I_{B}\left(t\right)$, which consists of piece-wise monotone functions, indicated by different colours and labelled by numbers from 1 to 10. This input is fed to the kernel operator $k_{p}$ with arbitrary selected values for $p_{1}$, $p_{2}$ and *a*. The result of $k_{p}$ is shown in Figure 6b. The colours in the plot correspond to the those of the piece-wise function in Figure 6a.

The KP model of hysteresis defined by Equation (13) can be interpreted as an infinite sum of weighted KP operators. This property simplifies the numerical implementation of the model, as the Preisach plane *P* can be divided into *L* regions, containing N=L(L+1)/2 KP operators, and the model can be described as a sum of *N* operators:

(18) $$b\left(t_{i}\right)=\sum_{j=1}^{L}\sum_{k=1}^{j}k_{p_{jk}}\left(I_{B}\left(t_{i}\right),\xi_{p_{jk}}\left(t_{i}\right)\right)\mu_{p_{jk}}+\varepsilon .$$

**Figure 7:**
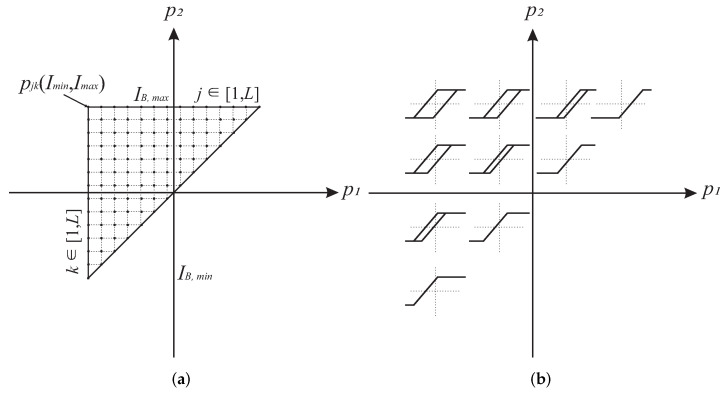
Preisach plane. (**a**) Discretization scheme of the Preisach plane. (**b**) Evolution of the shape of the KP operators on the Preisach plane.

*j* and *k* are the corresponding indices, and ε is the discretization error. The ridge rise factor *a* is heuristically determined as $a=\left(I_{B,\;max}-I_{B,\;min}\right)/\left(L-1\right)$ where $I_{B,\;min}$ and $I_{B,\;max}$ are the minimum and maximum operational EMBr current. Figure 7 shows the discretized Preisach plane and the shape progression of the KP operators in the Preisach plane.

The discrete model in Equation (18) can be written in matrix form as

(19) y=K·μ.

$K\in R^{Q\times N}$ is the kernel matrix that contains the information of the previous model state for the last *Q* samples of input values where i∈1,…,Q. $y\in R^{Q}$ is the output vector. $\mu \in R^{N}$ is a weight vector that is unknown and has to be identified. Weights that describe the system hysteresis can be obtained from the known input–output pairs by calculating

(20) $$\mu =K^{+}\cdot y,$$

where $K^{+}$ is the Moore–Penrose inverse of the kernel matrix [27].

### 3.2. Compensation for the Impact of the EMBr on the Flow-Induced Magnetic Field Measurements

In order to be able to use the KP model to model a process hysteresis, hysteresis has to satisfy two properties: the wiping-out property and the congruency property. The wiping-out property states that each local extremum wipes out the vertices prior to the extrema. The congruency property states that all hysteresis loops corresponding to the same extreme input values are congruent in the geometrical sense. These properties are shown to be satisfied for the hysteresis exhibited by ferromagnetic materials by Mayergoyz, and Krasnosel’skii and Pokrovskiǐ [24,28]. Under the assumption that the observed hysteresis of the in-phase magnetic field measurement is a direct result of the magnetic hysteresis of the ferromagnetic parts, for which the two properties are inherent, it follows that the same properties hold for the hysteresis observed in the in-phase magnetic field measurement. Thus, we can use the KP model to predict the compensation values for the offset of the measured in-phase magnetic field induced by the change in the EMBr current.

#### 3.2.1. Compensation via the Numerical Model of Hysteresis

**Figure 8:**
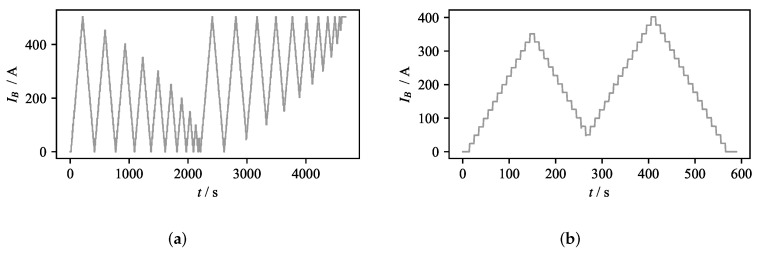
EMBr current profiles used for KP model training and validation. (**a**) Current profile used for identification of model weights. (**b**) Current profile used for validation of the model.

**Figure 9:**
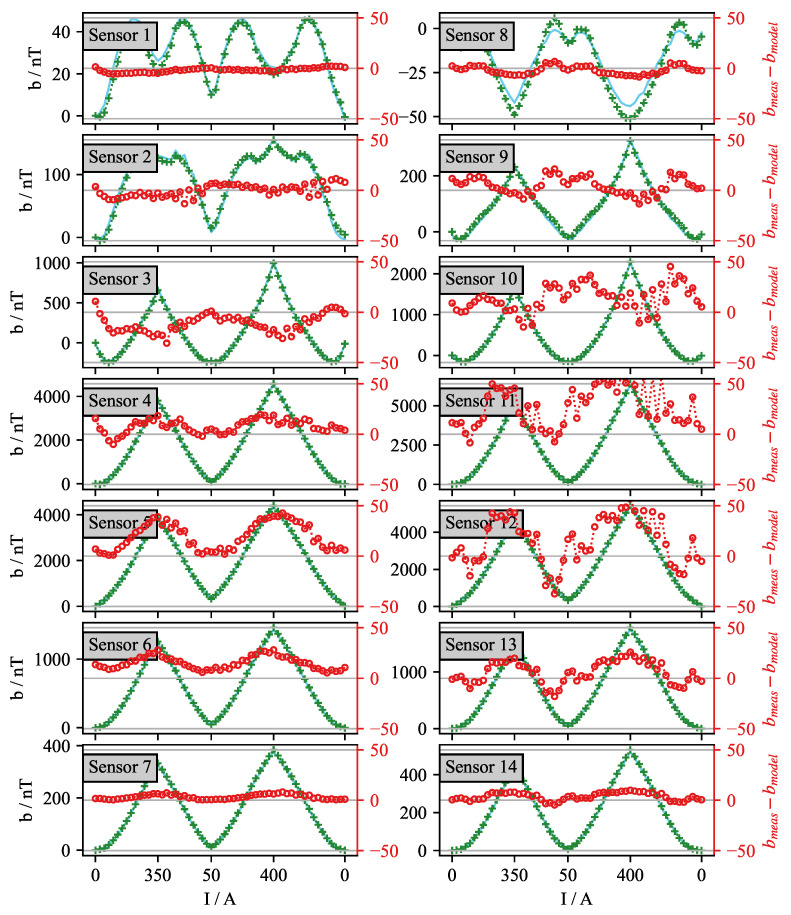
Plots of the absolute difference (red) between the measured in-phase magnetic field offset (green +) and the value predicted by the KP model of hysteresis (blue).

The compensation strategy for the real-time control was tested by implementing the numerical KP model of hysteresis. The Preisach plane was discretized with *L* = 25 levels, giving a total of *N* = 325 nodes for which the corresponding weights are identified. In order to identify the weights of the model, an identification experiment without flow is performed, for which an identification current profile is shown in Figure 8a. The identified model is validated by compensating for the effects of the current profile $I_{B}\left(t\right)$ shown in Figure 8b. The validation experiment was conducted without liquid metal flow, and the measured offset of the magnetic field was recorded. The comparison between the experiment and the output of the hysteresis model is given in Figure 9.

In these results, it can be seen that the numerical model has limited accuracy. Although the error relative to the maximum offset resulting from the EMBr influence is in the order of 1%, for specific sensors this translates to an absolute error larger than 50 nT, which is in the same range as the expected flow-induced field. Sensors with the highest error are positioned near the ferromagnetic yoke, and they experience the most considerable influence by the change in the EMBr current. The model’s error is a result of the limited capabilities of the EMBr current source, the limited accuracy of the current measurement performed with the current clamp and the temperature effects from the increased ohmic losses during the identification process. A precise current measurement could enable the hysteresis compensation before the signal demodulation to the in-phase and out-phase component of the magnetic field. This is one of the next steps in the improvement of the experimental setup.

#### 3.2.2. Congruency-Based Compensation

**Figure 10:**
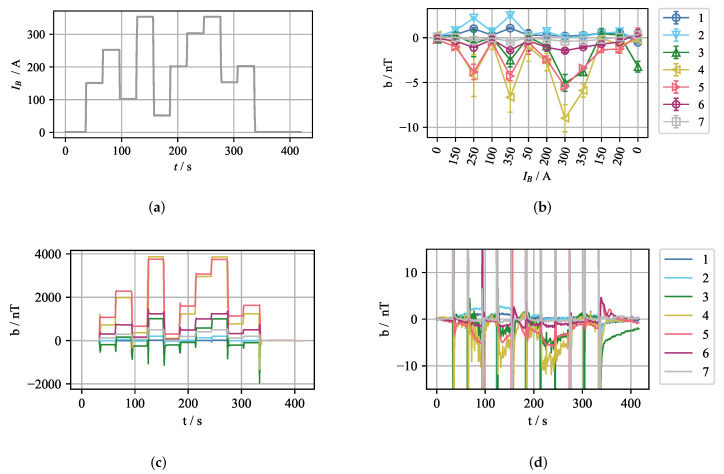
Congruency-based compensation for the impact of the EMBr current on the flow-induced magnetic field measured by sensors 1–7. (**a**) EMBr current profile. (**b**) Mean value and standard deviation of the magnetic field offset after the compensation for the periods between the current changes. (**c**) Uncompensated in-phase component of the magnetic field. (**d**) Compensated in-phase component of the magnetic field.

The inherent congruency property of the ferromagnetic hysteresis can be used for a simple compensation procedure. Proving that this property also holds for our case is straightforward. Across consecutive experiments, with the same EMBr current sequence, the current-offset value pairs should be the same. For two consecutive experiments without flowing liquid metal, the current was changed with the profile, as depicted in Figure 10a. The measurement offset for the first experiment is depicted in Figure 10c. Next, from this measurement the mean offset value for every current step was extracted and stored. These values were then used for compensation in the second experiment. The compensated magnetic field is depicted in Figure 10d, and the mean value and standard deviation after the compensation for the periods between the current changes is shown in Figure 10b. In this manner, very good compensation can be achieved. The error is less than 10 nT, with the highest error for sensor 4. A significant contribution to the error stems from the temperature increase in the ferromagnetic yokes due to the increased ohmic losses of the EMBr coils. However, the error range is acceptable even with the ohmic losses, and the temperature effects were not further considered in the compensation.

The congruency-based compensation method discussed so far is not optimal for real-time control because it is only viable for predetermined values of the EMBr current. This is because the compensation values for other values of the EMBr current cannot be precisely interpolated from the predetermined set of currents. Conventional control strategies do not impose this constraint on the controller. Instead, the controller’s output can take any value, between the minimum and maximum, based on the actual state of the flow in the mould. However, for the purpose of flow monitoring during regular operation, where the EMBr current is set based on the product recipes, or for a simple on/off controller, the congruency-based method is an optimal choice because of its accuracy and low identification time.

**Figure 11:**
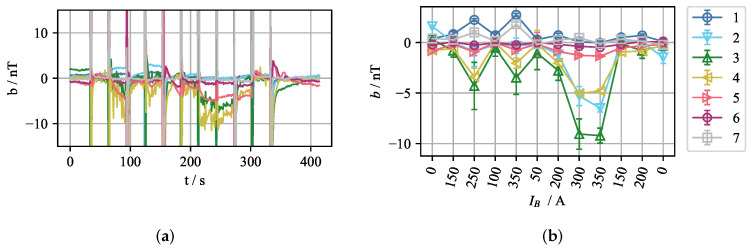
Results of congruency-based compensation for sensors 1–7, utilizing the numerical KP model of hysteresis for the experiment from Figure 10. (**a**) Time plot of the compensated in-phase component of the magnetic field. (**b**) Mean value and standard deviation of the compensated in-phase component of the magnetic field for periods in which the EMBr current is kept constant.

Considering the better accuracy of congruency-based compensation in contrast to the numerical model, it is the preferred initial choice. However, because the congruency property is inherent in the numerical KP model, the model itself can be utilized for the congruency-based compensation, provided that the weight identification is performed only for the expected EMBr currents. Figure 11 shows the congruency-based compensation by using the numerical model for the same previous scenario as in Figure 10. The overall performance of the numerical model is good and yields similar results as the initially described congruency-based compensation procedure.

The compensation procedures described in this section are an integral part of utilizing CIFT for process monitoring or in the feedback of the control loop. It needs to be very accurate so that the CIFT reconstruction can produce a realistic image of the flow because the controller decides on the subsequent actions based on this reconstruction. Significant compensation errors can lead to poor reconstruction quality, and a congruency-based compensation approach is pursued in the actual state of the experiment. Because the numerical model of hysteresis can perform in this mode well, it was implemented, and with further investigations to improve the model accuracy, the control strategy can be expanded.

The congruency-based compensation was used successfully in a simple control loop in the same experimental setup. The controller actuated the EMBr current to reject an artificial disturbance to the flow [22]. The controller was implemented for two values of the EMBr current, which were determined in advance. The congruency-based model yielded reliable predictions and could be easily and precisely trained.

The focus of this section was on compensating for the hysteresis effects. However, because this is a direct result of altered $B_{0}$, the effects of the EMBr on the reconstruction quality should be further investigated.

## 4. Real-Time Reconstruction

If CIFT should be used as a monitoring tool, or as a basis for process control, the flow reconstruction has to be performed in the same time frame when measurements of the flow-induced magnetic field are available. The solution of the regularized minimization problem of Equation (10) exploits the least squares method to delineate the following linear equation system [29]:

(21) $$\left({\bar{C}}^{\;T}\bar{C}+G_{\lambda_{G}}^{\;T}G_{\lambda_{G}}+L_{\lambda_{L}}^{\;T}L_{\lambda_{L}}+\lambda D^{\;T}D\right)\tilde{v}=A_{\lambda }\tilde{v}={\bar{C}}^{\;T}\bar{b}.$$

Here, the matrix $A_{\lambda }\in R^{3m\times 3m}$ is the sum of squares of the system matrix $\bar{C}$, the divergence matrix G, the Laplacian matrix L, and the regularization matrix D for a given regularization parameter λ. The solution to the linear inverse problem is obtained by using Cholesky decomposition. The optimal regularization parameter $\lambda_{opt}$ is selected via the L-curve method [30]. During this procedure, Equation (21) is solved for different values of λ using the computationally heavy Cholesky decomposition with $O({\left(3m\right)}^{3})$ complexity. The optimal regularization parameter is found at the maximum curvature of the L-curve [23]. Typically, Equation (21) has to be solved 20 to 40 times, until the best parameter is found. Even on a fast CPU or a GPU, the final solution takes from several seconds up to half an hour, depending on the mesh size. The reconstruction time can be significantly reduced if the matrix products

(22) $$F_{\lambda }=A_{\lambda }^{-1}{\bar{C}}^{\;T}$$

are precalculated for a predefined set of regularization parameters [31]. Then, the Cholesky decomposition is replaced by the matrix vector product

(23) $${\tilde{v}}_{\lambda }=F_{\lambda }\bar{b}.$$

This procedure reduces the number of operations to O(3m·n) with n≪3m. Even for a fine grid, the entire reconstruction with automatic search can be executed in less than one second [31] on a standard CPU. An additional speed-up is expected, if GPUs are used.

The presented procedure requires that the following two assumptions have to be fulfilled: (i) the optimal regularization parameter varies only in a narrow interval over the entire experiment and (ii) the reconstruction quality is only slightly diminished for regularization parameters in the neighbourhood of the maximum curvature of the L-curve.

In order to check if these assumptions are reasonable for the present application, and to investigate the quality of the real-time reconstruction, we started with a OpenFOAM transient flow simulation in the mould without an active EMBr. The inlet velocity at the SEN was set to 0.9 m/s, corresponding to the experimental conditions. The mesh consists of 5 × 10${}^{6}$ cells, and the pisoFoam solver with the omegaSST turbulence model was selected. The simulation was stopped at 100 s and served as a basis to solve the forward problem and calculate the magnetic field at the sensors for each time step. This simulated sensor signal is fed to the CIFT reconstruction solver and for each time step a reconstructed velocity field is obtained. By comparing the original and the reconstructed velocity field, the quality of the reconstruction can be assessed.

**Figure 12:**
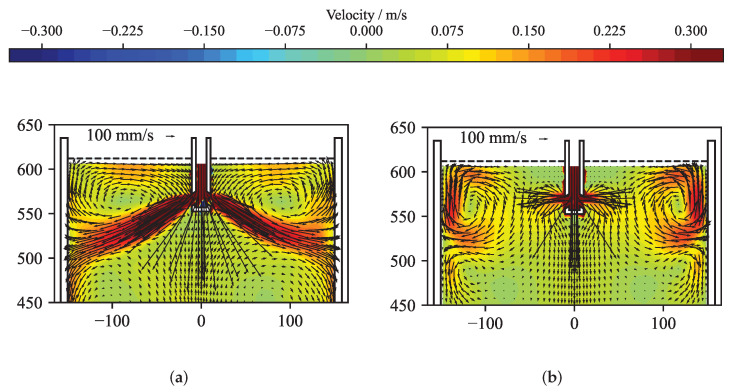
Reconstruction of the time dependant flow field at t= 17 s. (**a**) Three-second time average of the velocity field obtained from the numerical simulation. (**b**) Reconstructed velocity field for the optimal regularization parameter selected via the L-curve method.

**Figure 13:**
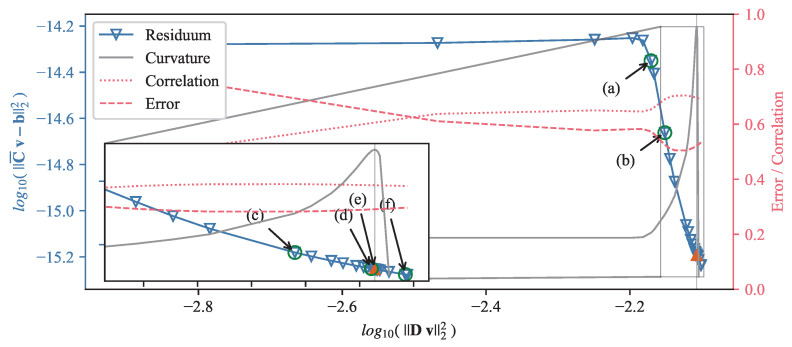
The L-curve for the Reconstruction of the time dependant flow field at t= 17 s. Annotated (a)–(f) are the regularization parameters for which the velocity field is shown in Figure 14. $\lambda_{opt}$ is marked by a red triangle and annotated with (e). (a) λ= 1.000 ×$10^{-8}$. (b) λ= 1.000 ×$10^{-9}$. (c) λ= 1.000 ×$10^{-10}$. (d) λ= 2.125 ×$10^{-11}$. (e) $\lambda_{opt}=$ 1.896 ×$10^{-11}$. (f) λ= 1.000 ×$10^{-12}$.

**Figure 14:**
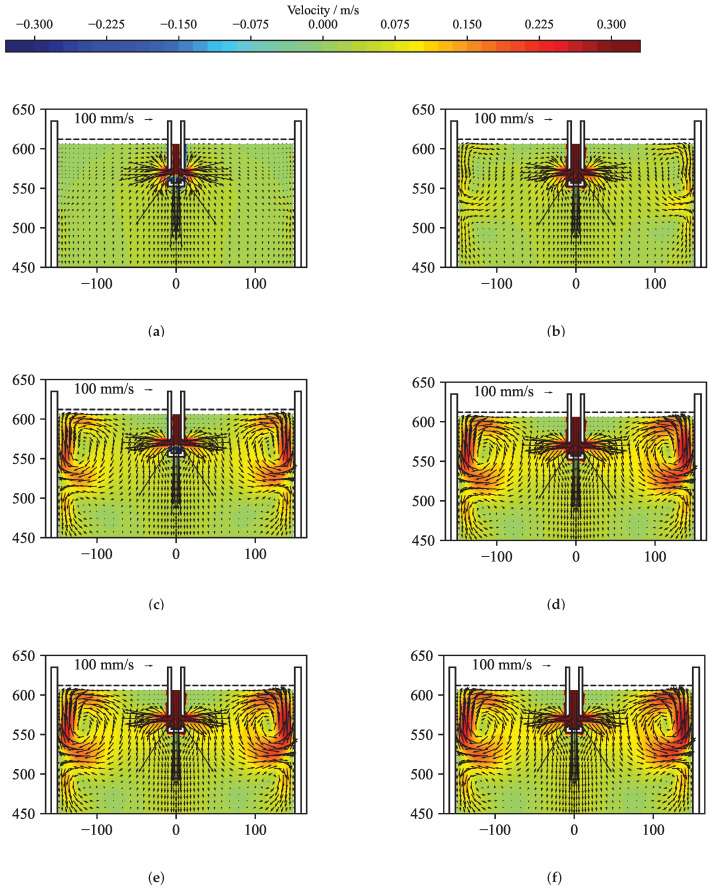
Velocity reconstructions for the regularization parameters annotated in Figure 13. (**a**) λ= 1.000 ×$10^{-8}$. (**b**) λ= 1.000 ×$10^{-9}$. (**c**) λ= 1.000 ×$10^{-10}$. (**d**) λ= 2.125 ×$10^{-11}$. (**e**) $\lambda_{opt}=$ 1.896 ×$10^{-11}$, the optimal regularization parameter selected via the L-curve method. (**f**) λ= 1.000 ×$10^{-12}$.

Figure 12 presents the original and the reconstructed velocity for a time averaged case. The solution of the reconstruction was performed using the standard solver. Figure 12a shows the velocity structure at the mid-plane of the mould obtained from the OpenFOAM simulation by averaging over 3 s. From this velocity field, the flow-induced magnetic field was calculated by Equation (8) and was randomly perturbed with values up to ±5 nT. Figure 12b shows the reconstructed velocity field in the mid-plane of the mould for the optimal regularization parameter. In comparison to the original velocity field in Figure 12a, the flow structure close to the narrow faces shows a reasonable agreement. With sensors only positioned at the narrow sides of the mould, the inverse problem shows a clear preference for reconstructing the velocities close to the narrow faces, while somehow suppressing, by virtue of the regularization, the more internal velocity. Figure 13 depicts the corresponding L-curve, curvature, correlation and mean quadratic error of the reconstruction from Figure 12. After the initial L-curve is calculated, the refinement algorithm is iteratively seeking the optimal regularization parameter at the point of the maximum curvature of the L-curve. A total of 31 regularization parameters were explored, showing that the curvature was highest for $\lambda_{opt}$ = 1.896 × 10${}^{-11}$. The error and correlation are calculated for the original and reconstructed velocity vector field only for the top part of the mould for z≥ 450 mm, because the magnetic field sensors are located in this region. In order to demonstrate that the correlation as well as the mean quadratic error change only very slightly in the neighbourhood of the maximum of the curvature, six different regularization parameters were selected and annotated in Figure 13. For all six regularization parameters, the key metrics are shown in Table 1 and the reconstructed mid-plane velocity field are presented in Figure 14. The corresponding differences are barely noticeable, and the correlation and the error for the reconstructions (d) and (f) differs less than 1% from the ones for the optimal regularization parameter of the reconstruction (e). Therefore, assumption (ii) is fulfilled.

**Figure 15:**
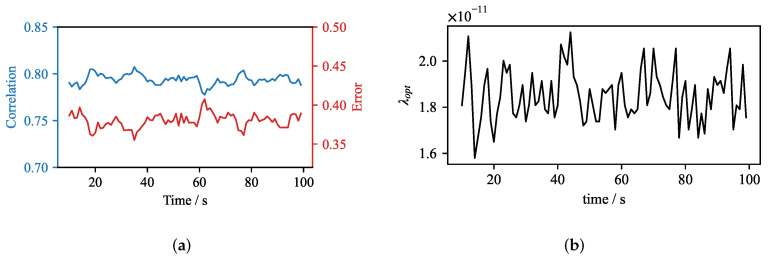
Time evolution plots of error, correlation and optimal regularization parameter. (**a**) Error and correlation plot of reconstructions for optimally selected regularization parameter $\lambda_{opt}$. (**b**) Temporal evolution of the optimal regularization parameter $\lambda_{opt}$.

In order to investigate the time evolution of the reconstruction, we reconstructed the time dependent velocity structure from the OpenFOAM simulation using the standard inverse CIFT solver for every second in the interval between 20 s and 100 s. The flow was averaged with a 3-s moving average in order to remove the small eddies which are ambiguous to reconstruct. The time evolution of the correlation and the error of the reconstructed velocity are shown in Figure 15. The correlation of the reconstruction fluctuates around 0.8, and the error varies slightly below 0.4, which is reasonable for the present configuration.

The according values of $\lambda_{opt}$ are presented in Figure 15b. The optimal parameter is found within a very narrow range of values between 1.6 × 10${}^{-11}$ and 2.1 × 10${}^{-11}$. This result indicates that assumption (i) holds. Therefore, a precise reconstruction should be possible using the real-time reconstruction, if this interval is sampled sufficiently. Therefore, we selected a subset of 22 regularization parameters common across all time steps of the reconstruction and reconstructed the flow field in the mould using the real-time procedure.

**Figure 16:**
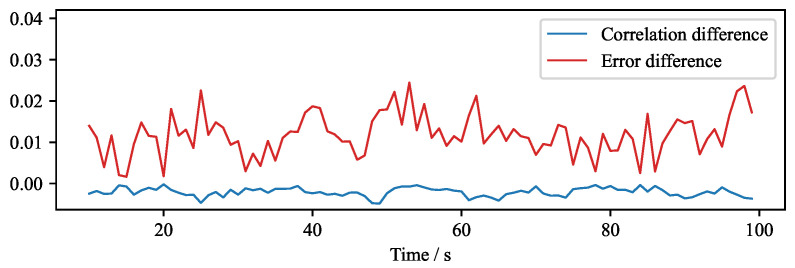
Absolute difference of error and correlation between the velocity fields reconstructed by the standard and the real-time algorithm.

The impact of the real-time algorithm on the quality can be quantified by comparing the reconstructions which use the predefined set of regularization parameters with the reconstructions obtained by the original fine search of the regularization parameter. Figure 16 shows the absolute difference in the correlation of two reconstruction approaches. It can be seen that the absolute difference of the correlation is small, and the maximum error difference is close to 2%.

**Figure 17:**
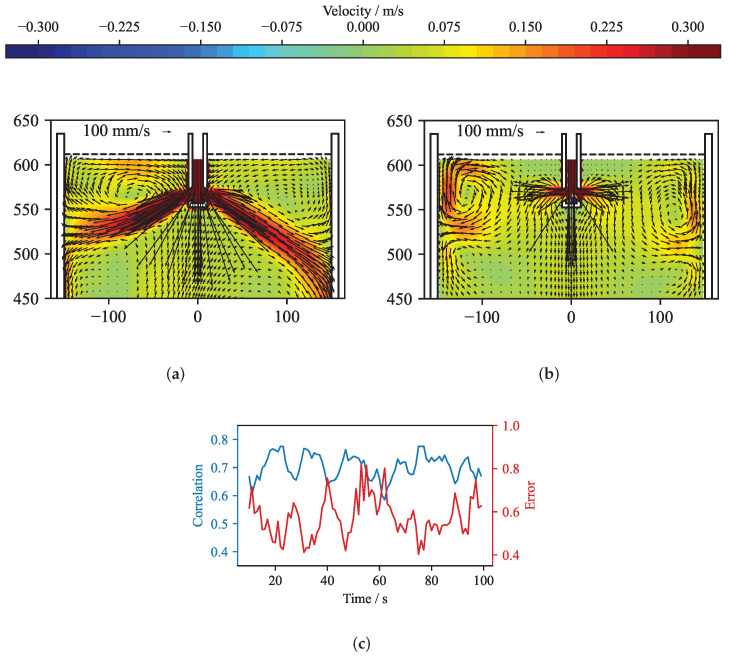
(**a**) Three-second time average of the velocity field obtained from the numerical simulation at t= 40 s. (**b**) Reconstructed velocity field. (**c**) Time plot of error and correlation for velocities close to the narrow face of the mould. |x|≥ 100 mm. *y* = 0 mm, z≥ 450 mm.

To calculate the error and the correlation in Figure 13 and Figure 15a, only the velocities from the top section of the midplane of the mould were used. However, if just the mid-plane velocities closest to the narrow faces of the mould (|x|≥ 100 mm) are observed, the correlation drops and the error increases as shown in Figure 17. This slight deterioration results from the slightly exaggerated magnitude and the smaller size of the top vortices. The peaks in error and dips in correlation correspond to the time periods in which the jet impinges deeper on one side, associated with the fact that the velocities in the top roll decrease, and the vortex changes the shape. This is clearly seen for the time *t* = 40 s, as shown in Figure 17a,b. Figure 17a shows the time average velocity field obtained from the numerical simulation and Figure 17b depicts the reconstruction of the same velocity field.

**Figure 18:**
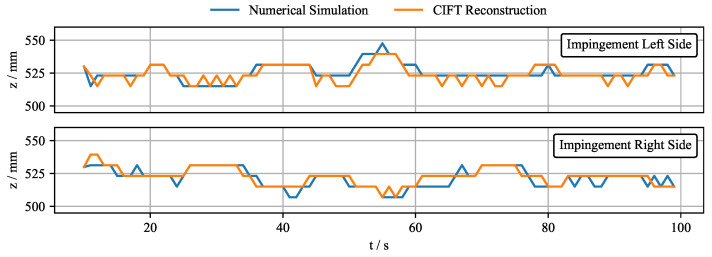
Comparison of the jet impingement point obtained from the numerical simulations and the CIFT real-time reconstruction for the same numerical velocity field.

Nonetheless, it is possible to use the real-time reconstruction algorithm for control if a key feature of the flow can be observed. For this purpose, the jet impingement position is selected. It is a crucial parameter that can be reliably extracted and easily controlled with the EMBr and from which information about the flow structure can be inferred. Figure 18 shows the comparison of the impingement position from the numerical simulation and the CIFT real-time reconstructions of the same velocity field. It can be seen that CIFT can reconstruct the impinging position very well. The error is only a few millimetres, which corresponds to the discretization size of the mesh.

Overall, the quality of the real-time reconstruction is not hindered by the fact that only a predetermined set of regularization parameters is being evaluated and that the inverse of the system matrices is precalculated. The key flow features can be reliably extracted, and the CIFT fast reconstruction algorithm can be used for real-time monitoring and control.

An earlier publication considered the flow control based on the actual jet impingement point derived from the CIFT reconstruction [22]. An obstacle was added at one of the SEN outlets, which deflected the jet and reduced the flow on that side. The flow asymmetry was further exaggerated when the EMBr current was set to 200 A. CIFT was able to reconstruct the flow in real-time and based on the reconstructions, the controller successfully detected the change in the jet impingement point and changed the EMBr current set-point to 0 A.

## 5. Conclusions

This publication addresses two critical prerequisites for implementing CIFT in continuous casting, either as a monitoring tool or as feedback for the control: (i) compensation for the impact of the strong magnetic field of the EMBr, and (ii) real-time reconstruction of the velocity field. It is shown that CIFT now satisfies the two requirements and is able to provide additional information on the status of the process, thus paving the way for further process improvement. Furthermore, the reconstructions can reliably provide the position where the jets impinge on the narrow faces of the mould, a vital feature of the flow that could be effectively controlled by the EMBr. The effect of the EMBr on the measurement system is challenging for the case where the EMBr current is unknown prior to the experiment. However, for the case where the operating parameters of the EMBr are known well in advance, the compensation can be performed successfully, enabling simple control loops that rely on CIFT as shown in an earlier publication [22].

Applying more complex control strategies, rather than just controlling the jet impingement position, requires additional validation of the flow reconstruction from the mini-LIMCAST and identification of additional key features that can be controlled. Ultrasound Doppler Velocimetry (UDV) is a well established measurement method for liquid metals which can be used to measure the actual flow in the mould. However, applying it in combination with CIFT in the current configuration presents a challenge. During the experiments, the highly turbulent flow will generate a significant amount of gallium-oxide, which increases the attenuation of the ultrasound beam, requiring replacement or cleaning of the liquid metal. Additionally, because the UDV can measure only the velocity component in the direction of the ultrasound beam, and the CIFT coils are positioned on the narrow faces of the mould, the UDV measurements can only be performed from the top, providing only the vertical velocity component. Alternatively, the UDV and CIFT measurement can be performed independently, and comparison of the average flow field can be performed. These, and many more considerations, have to be made in order to properly validate the CIFT reconstructions in the mini-LIMMCAST mould, which is one of our immediate research goals.

Moreover, future research should build on the results presented in this publication and focus on implementing a simple controller to optimize the jet impingement position for predefined sets of EMBr current values. Additional research should be devoted to developing more complex compensation models, possibly with a data-driven approach. Furthermore, some effort should be invested in the everlasting goal of improving the reconstruction quality. Finally, significant effort should be made in pursuing more complex control strategies.

## Figures and Tables

**Table 1:** Reconstruction parameters λ and corresponding values of error and correlation for the velocity reconstructions in Figure 14. Reconstruction (e) is done with the optimal regularization parameter calculated by the L-curve method.

Reconstruction	λ	Correlation	Error
(a)	1.000 × $10^{-8}$	0.65378	0.57316
(b)	1.000 × $10^{-9}$	0.69040	0.52493
(c)	1.000 × $10^{-10}$	0.70435	0.50469
(d)	2.125 × $10^{-11}$	0.69651	0.52070
**(e)**	**1.896 × $10^{-11}$**	**0.69601**	**0.52175**
(f)	1.000 × $10^{-12}$	0.69084	0.53272

## Data Availability

Data supporting the reported results is available as open access doi:10.14278/rodare.1641 (accessed on 26 March 2022).
